# Generation and modification of human locomotor EMG activity when walking faster and carrying additional weight

**DOI:** 10.1113/EP092063

**Published:** 2025-03-06

**Authors:** Bridgette A. P. Damewood, Thomas Sinkjær, Aiko K. Thompson

**Affiliations:** ^1^ Department of Health Sciences and Research, College of Health Professions Medical University of South Carolina Charleston South Carolina USA; ^2^ Department of Rehabilitation Medicine Emory University School of Medicine Atlanta Georgia USA; ^3^ Department of Health Science and Technology Aalborg University Aalborg Denmark

**Keywords:** H‐reflex, loading response, locomotion, muscle afferents, walking speed

## Abstract

In activities of daily living, people walk at different speeds with or without carrying additional loads. In this study, we sought to examine how human adults manage these commonly encountered additional demands during walking. We measured electromyography (EMG), triceps surae H‐reflexes, joint motion and ground reaction forces (GRF) while participants walked at 1.0 m/s and 1.5 m/s with or without an additional 20.4 kg of weight (the equivalent of 23–36% bodyweight). Faster walking was accompanied by a universal increase in burst EMG amplitude across flexors and extensors of upper and lower leg muscles (with most notable increases found in the plantarflexors) while burst patterns of activity were maintained. In addition, the range of motion increased at the ankle, knee and hip joints, while the step cycle duration was shortened. In bearing additional weight, upper and lower leg extensor activity, especially early stance quadriceps activity, was increased while joint motion was minimally affected at the ankle and knee (but not hip). When walking faster and carrying additional weight, changes in locomotor EMG (except for plantarflexors) and knee and hip joint motion displayed combined features of those two additional demands; changes in plantarflexor activity and ankle joint motion were more complex. Locomotor H‐reflexes were larger at 1.5 m/s than at 1.0 m/s only when carrying additional weight. In generating plantarflexor activity and controlling ankle joint motion for propulsive force generation when walking faster and carrying additional weight, multiple mechanisms of both spinal and supraspinal origin may be involved.

## Abstract (highlights)

- **What is the central question of the study?**
  How do people walk faster and carry additional weight?
- **What is the main finding and its importance?**
  When walking faster, activity increases in ankle and knee flexors and extensors with little change in pattern; mid–late stance ankle extensor increases strikingly. Carrying additional weight increases extensor but not flexor activity. Walking faster while carrying additional weight additively alters knee and hip flexor/extensor activity and knee and hip motion but not ankle extensor activity or H‐reflexes. The findings support the interpretation of combined central and afferent input to shape and drive the spinal locomotor network.

## 1 INTRODUCTION

In activities of daily living, people walk at different speeds with or without additional load to be carried. In general, when walking speed increases, the amplitude of leg muscle activity increases at a specific phase of the gait cycle during which a given muscle is most active (Hof et al., 2002; Murray et al., 1984; Nilsson et al., 1985). For example, quadriceps activity increases primarily in the early stance phase; triceps surae activity increases primarily at the end of the stance phase (i.e., push‐off); and tibialis anterior (TA) activity increases primarily just before and during heel contact. Joint motion also becomes more robust at higher speeds (Fukuchi et al., 2018; Murray et al., 1984; Nilsson et al., 1985; Stoquart et al., 2008; Winter, 1983a) and temporal parameters of the gait (e.g., stance and swing duration, cadence) change (Andriacchi et al., 1977; Capaday & Stein, 1986; Cronin et al., 2013, 2009; Griffin et al., 2003; Hof et al., 2002; LaFiandra et al., 2003; Murray et al., 1984; Nilsson et al., 1985; Orendurff et al., 2004; Stoquart et al., 2008; Sun et al., 2018; van Hedel et al., 2006; Winter, 1983a). In terms of gait kinetics and kinematics, faster walking results in greater peak vertical and anterior–posterior ground reaction force (GRF) during the breaking and propulsion phases of stance (Andriacchi et al., 1977; Fukuchi et al., 2018; Sun et al., 2018), and greater rate of vertical GRF development at the beginning (Cook et al., 1997; Sun et al., 2018) and end (i.e., faster unloading) of stance (Cook et al., 1997). It appears that faster walking entails greater muscle activation, more robust joint motion, and enhanced force generation, yet the relative timing and features of key events (such as push‐off activity from plantarflexors and peak knee flexion) remain consistent across different walking speeds (Hof et al., 2002; Nilsson et al., 1985; Winter, 1983a; Yang & Winter, 1985).

When walking with the additional weight, whether the weight is uniformly distributed about the hip or trunk near the body's centre of mass or placed on the front or back of the body may have some effects on EMG activity, but the common observation is that there are clear increases in the duration and amount of ankle and knee extensor activity (Grey et al., 2002; McGowan et al., 2008; Stephens & Yang, 1999) but not in the flexor activity (Grey et al., 2002; Stephens & Yang, 1999) or ankle and knee joint motion (McGowan et al., 2008; Stephens & Yang, 1999). When additional weight is loaded on the back (e.g., as in wearing a backpack) or front of the body, trunk flexion changes (Fiolkowski et al., 2006; Krupenevich et al., 2015; LaFiandra et al., 2003; Liew et al., 2016; Simpson et al., 2011b; Wang et al., 2023) and hip and ankle range of motion and cadence increase (LaFiandra et al., 2003; Liew et al., 2016; Wang et al., 2023) with longer double support phase and shorter stride length (Gill et al., 2016; Huang & Kuo, 2014; Krupenevich et al., 2015; LaFiandra et al., 2003; Liew et al., 2016; Mexi et al., 2023; Simpson et al., 2012). Little is known about how additional weight on the front or back of the body affects locomotor EMG activity; one study reported increased EMG activity in the medial gastrocnemius and vastus lateralis (VL) but not in the ankle or knee flexors with increasing weight with backpack loading (Simpson et al., 2011a). In sum, changes in EMG, kinematics and temporal features of gait brought about by walking faster differ from those caused by carrying additional weight, suggesting that separate neural control mechanisms might be involved in these two types of common additional demands during walking.

Generating propulsive force is essential in walking, and ankle plantarflexors are primary contributors to propulsive force generation (Griffin et al., 2003; Kulmala et al., 2016; Neptune et al., 2001, 2008; Orlovsky et al., 1999; Simonsen, 2014; Winter, 1983a). During the push‐off phase (i.e., end of stance), the ankle plantarflexors, soleus and gastrocnemii, are estimated to produce a total of 85% (60% and 25%, respectively) of propulsive force (Neptune et al., 2001). Proprioceptive afferents contribute to activation of plantarflexor muscles during this phase of walking (for reviews see Bouyer, 2011; Donelan & Pearson, 2004b; Frigon et al., 2021; Lam & Pearson, 2002). In cats, load sensitive Ib afferents from Golgi tendon organs fire during stance when the muscle is loaded (Donelan & Pearson, 2004a), likely contributing to the plantarflexor activity in this phase. Ia and II muscle spindle afferents from the ankle plantarflexors are thought to contribute to early stance phase of plantarflexor activity when muscle length is rapidly changing (Donelan & Pearson, 2004a; Prochazka & Gorassini, 1998; Prochazka et al., 1989). In humans, muscle afferents Ia, II and Ib, contribute differently to the stance phase soleus activity (af Klint, Mazzaro, et al., 2010; Grey et al., 2004, 2007; Mazzaro et al., 2005; Sinkjaer et al., 2000; Stephens & Yang, 1999). The Ia afferents may contribute to soleus activation as the ankle deviates unexpectedly from the natural trajectory of dorsiflexion (i.e., during the eccentric stretch of the soleus) (Mazzaro et al., 2005; Yang et al., 1991). Length‐sensitive group II spindle afferents are also thought to play a part in amplitude modulation of stance phase soleus activity (Mazzaro et al., 2006). Load‐sensitive group Ib afferents are suggested to contribute significantly to the locomotor soleus EMG (af Klint, Mazzaro, et al., 2010; Sinkjaer et al., 2000), in particular, enhancing the soleus activation in late stance (Grey et al., 2007). Cronin et al. (2009) found that at faster walking speeds, the larger soleus burst amplitude is accompanied by a reduced rate and amount of soleus fascicle length change in mid‐stance, suggesting that the feedback from muscle spindle afferents is an unlikely input source for increasing push‐off EMG activity that would support faster walking. (Currently, whether the fusimotor system regulates or increases the spindle sensitivity during human locomotion is unknown.) Even if the spindle afferent firing does not decrease, their effectiveness in exciting motoneurons through reflex pathways may be reduced when walking faster; Edamura et al. (1991) showed a few individual examples in which the soleus H‐reflex gain decreased with increasing walking speed. Similarly, adding extra weight does not affect locomotor stretch reflexes (Grey et al., 2002). Altogether, an emerging picture is that walking situations in which the demand for propulsive force generation increases, the ankle plantarflexor activity increases in mid–late stance (Griffin et al., 2003; Hof et al., 2002; Kulmala et al., 2016; Neptune et al., 2001, 2008; Winter, 1983a) and muscle afferents (but not necessarily Ia afferents) are likely contributors to increasing plantarflexor activity (af Klint et al., 2010b; Grey et al., 2004, 2007; Mazzaro et al., 2005; Sinkjaer et al., 2000; Stephens & Yang, 1999). What is yet to be understood is how similarly or differently these afferents may contribute to walking faster and carrying additional weight, both of which increase the demand for propulsive force generation.

Thus, in this study, we aimed to examine the neuromuscular strategies healthy adults utilize to walk faster and carry additional weight. Specifically, we asked the participants to walk at two distinct speeds (i.e., 1.0 m/s, a speed close to their self‐selected walking speed, and 1.5 m/s, a speed that was clearly faster than 1.0 m/s) while bearing an additional fixed weight of 20.4 kg (the equivalent of 23–36% bodyweight) and measured EMG activity in upper and lower leg muscles, soleus and lateral gastrocnemius (LG) H‐reflexes, joint kinematics, GRF, and temporal parameters of gait. Women of similar physical size were studied so that the two walking speeds and the fixed amount of additional weight would have a similar impact on gait across participants. Further, to aid the interpretation of the push‐off phase plantarflexor activity, the H‐reflexes, ankle rotation and GRF development were also analysed for the mid–late stance phase of walking.

## 2 METHODS

### 2.1 Ethical approval

The study protocol was approved by the Institutional Review Board of the Medical University of South Carolina, Charleston, SC, USA (Protocol 00042824). It complied with the ethical guidelines of the *Declaration of Helsinki* (except for registration in a database). All participants gave written informed consent before participating in the study.

### 2.2 Participants

Eleven physically active healthy adult women (27.4 ± 5.8 (SD) years of age, 163.4 ± 3.9 cm in height, 65.6 ± 9.3 kg in weight) with no known neurological or chronic orthopaedic injuries to their lower extremities participated in this study. For the present group of participants, 1.0 m/s was close to their self‐selected comfortable walking speed and 1.5 m/s was a manageable but clearly faster walking speed. The additional 20.4 kg corresponded to 23–36% of participants’ body weight, which was similar to the loading amount used in the previous study by Stephens & Yang (1999). Additionally, to examine the acute effects of carrying additional weight on the front or back of the body, individuals with a history of childbearing or individuals who were uncomfortable wearing the weighted vest and belt were not included in this study.

### 2.3 General procedures

**FIGURE 1:**
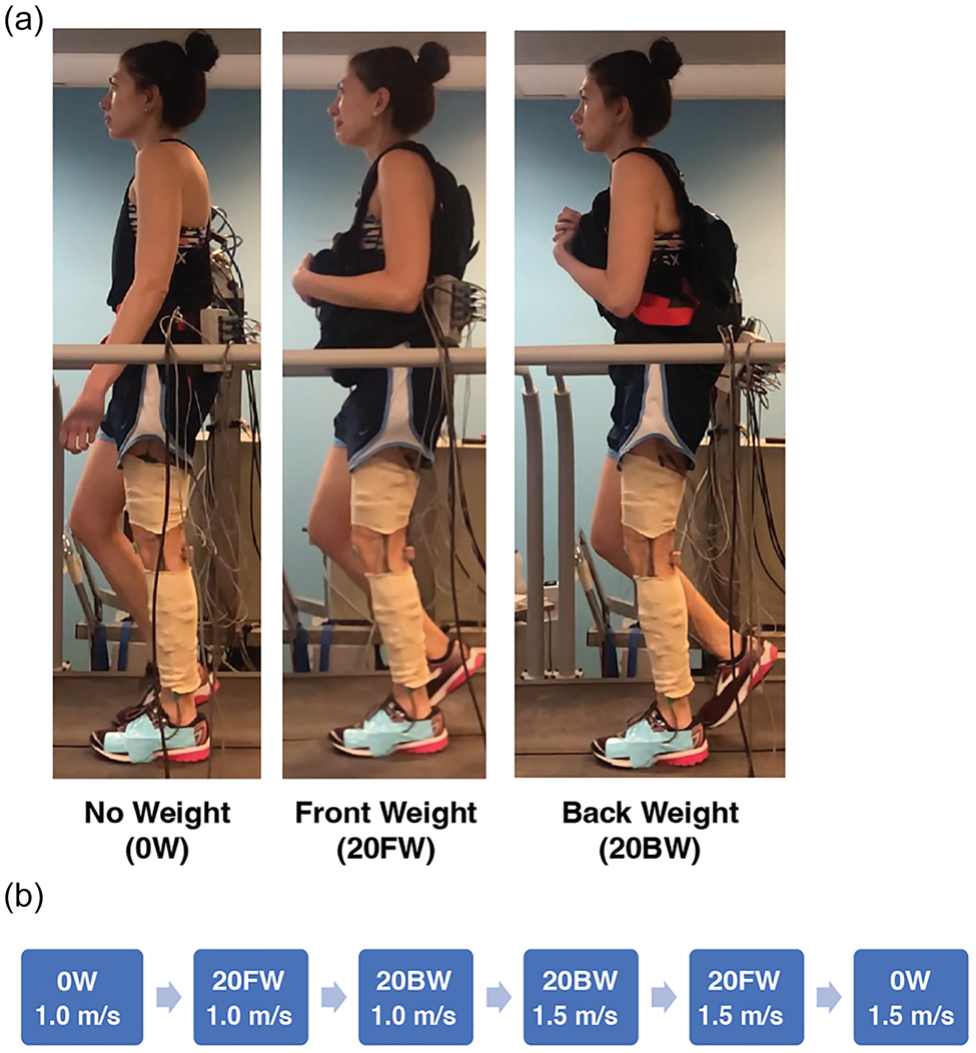
Protocol overview. (a) Experimental set‐up of weight conditions showing posture during mid‐stance of walking in one participant during the no additional weight (no weight, 0W) condition, and the 20.4 kg additional weight conditions onto the front (front weight, 20FW) and back (back weight, 20BW) of the body. (b) Order of speed and weight conditions that were used for all participants.

All measurements were made in a single experiment. For each participant, after the weighted vest and belt set (MIR Vest, San Jose, CA, USA) used for adding the 20.4 kg of weight was fitted for comfort, EMG recording and tibial nerve stimulating electrodes were placed on the left leg regardless of handedness or weight‐bearing tendency. Then, the tibial nerve was stimulated while the participant maintained her natural standing posture and corresponding level of soleus EMG activity (see EMG recording and electrical stimulation) to estimate the H‐reflex and M‐wave recruitment curves and the *M*_max_ in the soleus and LG. After stimulating the tibial nerve in standing, each participant walked on the treadmill at 1.0 and 1.5 m/s with or without an additional 20.4 kg of weight loaded entirely on the front or back of the body; two treadmill speeds (1.0 and 1.5 m/s) and three weight conditions (no additional weight (0W), front weight (20FW), and back weight (20BW)) were studied. For each of the six conditions, each participant walked for ∼10 min, while ankle, knee and hip joint motion, vertical and anterior–posterior ground reaction force (GRF_V_ and GRF_AP_, respectively), and locomotor EMG from multiple leg muscles were measured and the soleus and LG H‐reflexes were elicited (see EMG recording and electrical stimulation). In between conditions, the participant sat in a chair and rested for a few minutes. The following order of speed and weight conditions was used in all participants: 0W at 1.0 m/s, 20FW at 1.0 m/s, 20BW at 1.0 m/s, 20BW at 1.5 m/s, 20FW at 1.5 m/s, and 0W at 1.5 m/s (Figure 1). Immediately after the last bout of walking, the soleus and LG *M*_max_ were measured in standing, to confirm that EMG recording remained stable, and the extent of neuromuscular fatigue induced by 6 × 10 min of walking was minimal.

### 2.4 EMG recording and electrical stimulation

After cleaning the skin with isopropyl alcohol wipes and paper towels, surface self‐adhesive Ag–AgCl electrodes (2.2 × 3.3 cm; Vermed Inc., Nissha Medical Technologies, Buffalo, NY, USA) were placed for EMG recording over the soleus, LG, TA, biceps femoris (BF), vastus medialis (VM), VL and rectus femoris (RF) muscles over the muscle belly, with centres of electrodes ∼3 cm apart. For the soleus, electrodes were placed below the gastrocnemii and in line with the Achilles tendon. All EMG signals were amplified, band‐pass filtered at 10–1000 Hz (AMT‐8, Bortec Biomedical, Calgary, AB, Canada), and stored at the sampling rate of 4 kHz (Axon Digidata 1440A, Molecular Devices, San Jose, CA, USA).

To elicit the H‐reflex and M‐wave in the soleus and LG, the tibial nerve was stimulated in the popliteal fossa with single 1‐ms square pulses using a Digitimer DS7A constant current stimulator (Digitimer Ltd, Welwyn Garden City, UK) and delivered through a pair of surface self‐adhesive Ag–AgCl electrodes (2.2 × 2.2 cm for cathode and 2.2 × 3.3 cm for the anode; Vermed). For each participant, the locations of nerve‐stimulating electrodes were optimized to minimize the H‐reflex threshold and to avoid stimulation of other nerves. Before the first bout of walking (i.e., 0W at 1.0 m/s), to estimate the H‐reflex and M‐wave recruitment curves (to help determine the M‐wave amplitude to be used for the subsequent locomotor H‐reflex assessments) and obtain the maximum M‐wave (*M*_max_) in the soleus and LG, the tibial nerve was stimulated when the participant had maintained her natural standing posture and corresponding level of soleus EMG activity without additional weight. For this measurement, the background EMG was typically about 20 µV (in absolute amplitude, range 12–27 µV) for the soleus and 6 µV (range 5–9 µV) for the antagonist TA. Stimulus intensity was gradually increased from below the soleus H‐reflex threshold to just above the stimulus level at which the *M*_max_ was achieved. Four trials were averaged at each stimulus intensity.

To elicit the H‐reflex and M‐wave during walking, the tibial nerve was stimulated pseudo‐randomly throughout the step cycle with a minimum of one unstimulated step between stimuli (∼250 stimulated steps for each condition). To obtain the H‐reflexes with M‐waves of similar amplitudes across the step cycle, several different stimulus intensities were used for each speed/weight condition of walking (Capaday & Stein, 1986; Edamura et al., 1991; Llewellyn et al., 1990; Makihara et al., 2012).

### 2.5 Joint motion and GRF

Ankle, knee and hip joint motion in the sagittal plane was collected using electrogoniometers (Biometrics Ltd, Ladysmith, VA, USA) placed on the lateral surface of the leg or foot with the sensor crossing the axis of joint rotation. For the ankle, the two bases were aligned to the fibula and the fifth metatarsal, placed parallel to the ground, and crossing the lateral malleolus. For the knee, the two bases were aligned over the lateral femur and lower leg crossing the lateral femoral epicondyle. For the hip, the bases were aligned with the trunk and the lateral thigh.

For GRF measurements, GRF_V_ and GRF_AP_ were obtained for the right and left legs separately from the force plates instrumented onto the split‐belt treadmill (Bertec Corporation, Columbus, OH, USA).

Together with the EMG, the goniometer signal and GRF signal were sampled and stored at 4 kHz using the Axoscope data acquisition system (Axon Digidata 1440A, Molecular Devices).

### 2.6 Data analysis

Before performing any data analyses, first, to assess whether EMG recording remained stable and whether the extent of neuromuscular fatigue induced by 6 × 10 min of walking was limited (as intended), the amplitude of standing *M*_max_ measure before the first bout of walking was compared with the *M*_max_ measure after the last bout of walking. In all participants, the post‐walking *M*_max_ amplitude was within ±10% of the pre‐walking *M*_max_ (i.e., 4.8 ± 8.7% of the pre‐value). This would strongly support that over the course of experimental data collection, EMG recording remained stable, and neuromuscular fatigue was reasonably limited, and thereby the comparisons across different walking conditions would be valid.

For analysing locomotor data, the moment of heel contact was defined as the beginning of the step cycle (and thus, a step cycle was from one foot contact to the next foot contact by the same leg). The time of heel contact was detected as the earliest time instance at which point GRF_V_ exceeded 2 N (which was roughly equivalent to 1–1.5% bodyweight). For each participant's speed/weight condition, all steps were individually detected first, and then, all non‐stimulated steps (∼250 steps) were resampled to the mean step cycle duration (of non‐stimulated steps) and averaged together to generate the mean non‐stimulated step data. For the gait parameters described below, unless specified, all values were calculated on such mean non‐stimulated step data. Note that for each participant's each speed and additional weight combination condition, the quality, amplitude and amplitude modulation of all signals from all non‐stimulated steps were visually inspected for all steps; in no participant's no condition, we observed systematic (i.e., time‐related) declines or shifts in the obtained signals. This supports the likelihood that little fatigue or task‐adaptation was occurring during these data collections and thereby the validity of pooling and analysing ∼10 min of data together per condition.

#### 2.6.1 Temporal gait parameters

The end of the stance phase was determined as the time at which GRF_V_ returned to 0. The end of the swing phase was defined with the occurrence of heel contact (i.e., beginning) of the immediately following step. Based on the mean step cycle duration of the studied leg, calculated as described above, the stance and swing phase duration were expressed as a percentage of the step cycle duration.

#### 2.6.2 Joint motion

For each of the ankle, knee and hip joints, angles at heel contact, peak flexion and peak extension were calculated, and their timings in the step cycle were estimated and expressed as a percentage of the full step cycle. The median angle across the entire step cycle was also calculated for each joint. Specifically, for the knee joint, the angle and timing of peak knee flexion in the early stance (related to weight acceptance) phase were also calculated. In addition, to further the interpretation of propulsive force‐generating plantarflexor activity in the mid–late stance phase, ankle rotation amount and speed were calculated for 35–45% of the step cycle (i.e., mid–late stance phase); these measurements would serve as indirect measures of triceps surae muscle length change and its rate during this phase (af Klint, Cronin, et al., 2010; Grey et al., 2007).

#### 2.6.3 GRF

To estimate the propulsive force generated, we calculated the integral of the GRF_AP_ impulse from the studied side force plate over the entire propulsive phase (Balasubramanian et al., 2007). To further examine the potential afferent origins of plantarflexor activity in the mid–late stance phase, we also calculated the rate of force development (RFD) over 35–45% of the step cycle in GRF_V_ and GRF_AP_, respectively. These would serve as measures of loading that the muscles of the studied leg (including soleus and LG) were presumably experiencing during this phase.

#### 2.6.4 Locomotor EMG

To generate the averaged non‐stimulated locomotor EMG, the raw EMGs of unstimulated steps were rectified, resampled to the mean step cycle duration as mentioned above and averaged together (Kido et al., 2004b; Makihara et al., 2012; Thompson et al., 2019; Yang & Stein, 1990). For quantitative analysis, the step cycle was divided into 12 bins of equal duration and the average EMG amplitude was calculated for each bin. Then, for each participant, the soleus and LG EMG were expressed as a percentage of the *M*_max_ value measured during standing. For all other muscles, the average rectified EMG amplitude for each of the 12 bins was expressed as the percentage of the amplitude in the bin with the highest amplitude during walking at 1.0 m/s with no additional weight (i.e., 0W). To quantify the muscle activity for each muscle, EMG values from the bins in which the muscle is clearly active were averaged together. In addition, to further consider the potential origins of quadriceps activity (i.e., VM, VL, RF), we also compared the quadriceps EMG separately for the beginning of stance (i.e., bins 1 and 2) and end of swing phase (i.e., bin 12) across the speed and weight conditions.

#### 2.6.5 Locomotor H‐reflex

The soleus and LG H‐reflex and M‐wave amplitudes were measured as the peak‐to‐peak values in time windows determined for each participant. Typically, time windows of 29–45 ms and 5–22 ms post‐stimulus were used for the soleus H‐reflex and M‐wave, respectively. For the LG, 28–41 ms and 5–18 ms post‐stimulus were used for the H‐reflex and M‐wave, respectively. For comparing the reflex amplitudes across different phases of the step cycle, only the reflexes with a consistent amplitude of M‐wave (reflecting a consistent level of stimulation) were included for the locomotor H‐reflex analysis (Capaday & Stein, 1986; Edamura et al., 1991; Kido et al., 2004a; Makihara et al., 2012; Thompson et al., 2019). That is, some of the reflex responses with too large or too small M‐waves were eliminated from further analyses. In this study, H‐reflexes with the accompanying M‐wave amplitudes of 3–20% standing *M*_max_ were included in the final analyses for the soleus H‐reflex, and 3–38% standing *M*_max_ for the LG H‐reflex. Those H‐reflexes with similar M‐wave amplitude were then sorted into the 12‐step cycle bins of equal duration, averaged per bin and expressed in a percentage of the *M*_max_ that was measured during standing. To estimate the extent to which the H‐reflex was modulated during each walking condition, the modulation index was calculated as follows: 100 × [(highest bin amplitude − lowest bin amplitude)/highest bin amplitude] (Makihara et al., 2014; Zehr & Kido, 2001; Zehr & Loadman, 2012). In addition, to examine whether the phase in which the H‐reflex reached its peak amplitude differed across different speed and weight conditions, the bin with the largest H‐reflex amplitude was recorded for each walking condition in each participant. To further examine the potential afferent origins of plantarflexor activity in mid–late stance (i.e., propulsive force‐generating phase), the soleus and LG H‐reflexes in mid–late stance phase (i.e., bins 5 and 6) were averaged together and used to compare between speeds and weight conditions.

### 2.7 Statistical analysis

All data are presented as group means ± SD unless otherwise noted. To examine the effects of added weight and speed on locomotion, a two‐way (speed × weight) repeated measures analysis of variance (RM ANOVA) was applied to EMG amplitude (of selected bins per muscle), mid–late stance phase H‐reflexes, H‐reflex modulation indices, joint motion measures (i.e. angles at specific moments of the step cycle and their timings), GRF and temporal gait parameters. Mauchly's test was used to determine if the assumption of sphericity had been violated and where appropriate, degrees of freedom were corrected using the Greenhouse–Geisser method. A Bonferroni adjustment for multiple pairwise comparisons was used for all *post hoc* analyses. All statistical analyses were conducted using IBM SPSS Statistics Version 27 (IBM Corp., Armonk, NY, USA) with the significance level set at 0.05.

Below in Section 3, only the statistically significant differences or trends are reported. For both the ANOVA and *post hoc* analyses, all results left unmentioned imply that they were statistically not significant.

## 3 RESULTS

### 3.1 Effects of speed and weight on muscle activity

**FIGURE 2:**
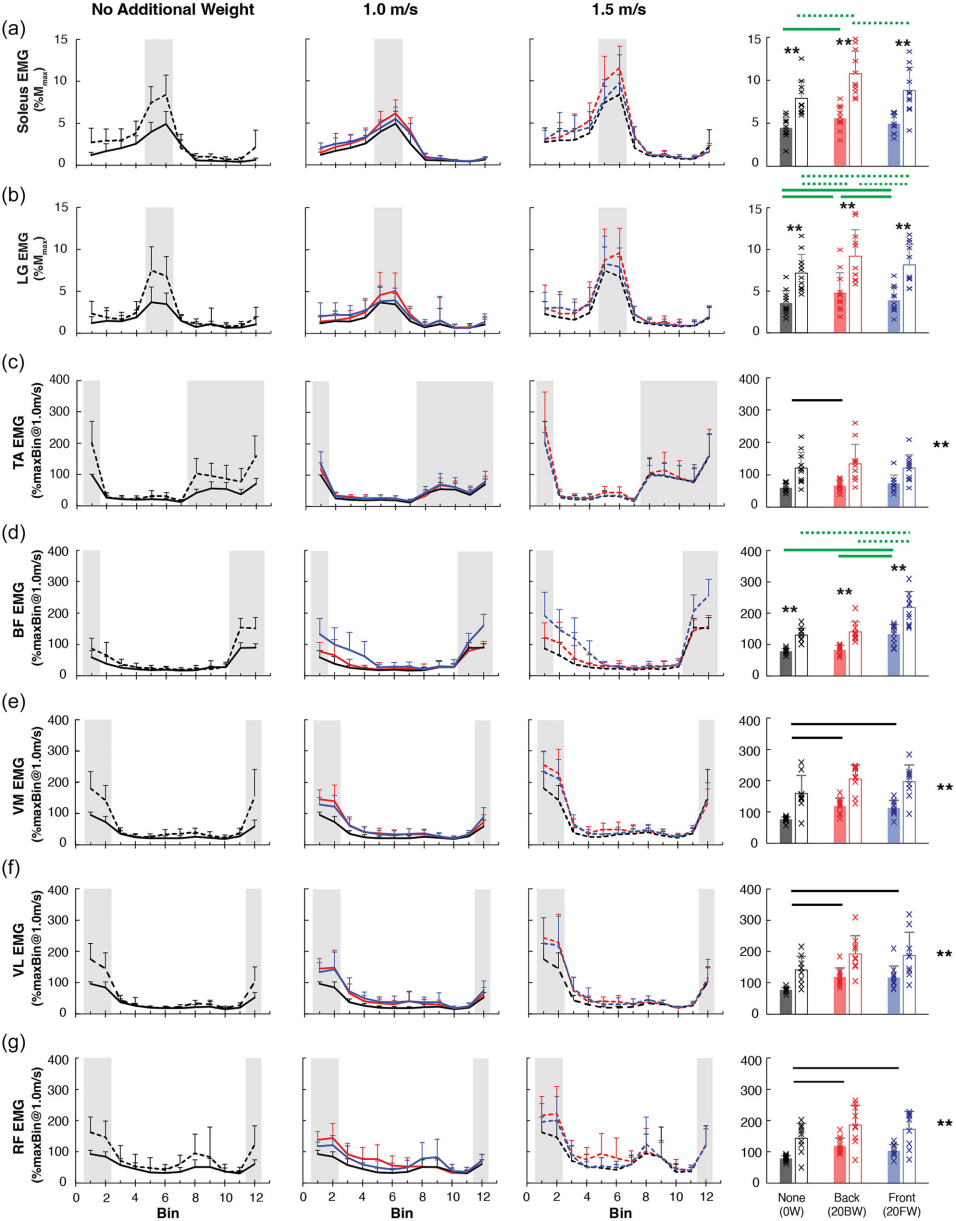
EMG activity in multiple leg muscles during walking at 1.0 m/s and 1.5 m/s with or without additional weight. Mean (+SD) locomotor EMG amplitude across the step cycle is displayed for soleus (a), LG (b), TA (c), BF (d), VM (e), VL (f), and RF (g). The step cycle was divided into 12 bins of equal duration and the mean EMG amplitude for each bin was calculated for each participant first, and then a group average was obtained. Continuous lines are for 1.0 m/s and dashed lines are for 1.5 m/s. Black lines are for no additional weight (0W), blue lines are for front weight (20FW) condition and red lines are for back weight (20BW) condition. For the soleus and LG (a, b), EMG amplitude is expressed as a percentage of the *M*_max_ measured during standing. For all other muscles (c–g), EMG amplitude is expressed as a percentage of the bin with the largest EMG amplitude (max bin) during walking at 1.0 m/s with 0W. For each muscle, the shaded phase of the step cycle indicates the bins averaged together to quantify the amount of muscle activity. Bar plots in the rightmost column summarize these values. Filled bars are for 1.0 m/s and open bars are for 1.5 m/s, with error bars indicating SD. Each ‘×’ symbol represents each individual participant's value. Asterisks to the right of the bars denote main effects of speed (i.e., different across all weight conditions). Asterisks above the bars indicate differences between 1.0 m/s and 1.5 m/s at a given weight condition (** *P *< 0.01, by paired comparison with Bonferroni correction). Black lines above the bars indicate differences between weight conditions and green lines above the bars indicate significant differences between the weight conditions: continuous lines for 1.0 m/s and dotted lines for 1.5 m/s (*P *< 0.05, by Bonferroni test). BF, biceps femoris; EMG, electromyography; LG, lateral gastrocnemius; *M*_max_, maximum M‐wave; RF, rectus femoris; TA, tibialis anterior; VL, vastus lateralis; VM, vastus medialis.

Locomotor EMG activity across 12 bins (group mean + SD) and the mean amplitude over a specific phase during which a given muscle is most active are shown in Figure 2 for each of the soleus, LG, TA, BF, VM, VL and RF muscles studied here. In general, for both the flexors and extensors, EMG activity over the individual muscle's active phase was larger at 1.5 m/s than 1.0 m/s (left column of Figure 2) across all seven muscles examined, similar to the previous reports (Hof et al., 2002; Murray et al., 1984; Nilsson et al., 1985). EMG activity in the extensors was larger when the participants carried additional weight than when the participants walked with no additional weight, in a phase‐specific manner (see middle two columns of Figure 2).

The two‐way repeated measures ANOVA revealed a significant main effect of speed for soleus (*F*_(1,10) _= 78.6, *P *< 0.001), LG (*F*_(1,10) _= 104, *P *< 0.001), TA (*F*_(1,10) _= 37.8, *P *< 0.001), BF (*F*_(1,9) _= 98.9, *P *< 0.001), VM (*F*_(1,8) _= 54.1, *P *< 0.001), VL (*F*_(1,8) _= 34.3, *P *< 0.001) and RF (*F*_(1,8) _= 21.3, *P *= 0.002), indicating larger EMG amplitude with 1.5 m/s than 1.0 m/s for all seven muscles. Across the seven muscles, EMG amplitude was 56–132% larger at 1.5 m/s than 1.0 m/s, (*P *< 0.01 for each of seven). The ANOVA also revealed a significant main effect of weight for soleus (*F*_(2,20) _= 36.7, *P *< 0.001), LG (*F*_(2,20) _= 17.4, *P *< 0.001), TA (*F*_(2,20) _= 4.6, *P *= 0.02), BF (*F*_(1.29,11.6) _= 28.3, *P *< 0.001), VM (*F*_(2,16) _= 23.7, *P *< 0.001), VL (*F*_(2,16) _= 17.5, *P *< 0.001) and RF (*F*_(2,16) _= 22.5, *P *< 0.001). The *post hoc* comparisons between weight conditions revealed that EMG amplitude was larger with the added weight conditions (20FW and 20BW) than the no additional weight (0W) condition for all extensor muscles (soleus, LG, VM, VL, RF): the quadriceps EMG (VM, VL, RF) was larger with 20FW and 20BW than with 0W (24–43% larger, *P *< 0.01 for all). For the soleus, EMG was 32% larger for 20BW (*P *< 0.001) and 11% larger for 20FW when compared to 0W (*P *= 0.02) and was largest for 20BW when compared to 20FW (18% larger, *P *= 0.003). For the flexor muscles (TA and BF), the TA EMG was ∼10% larger with 20BW than with 0W (*P *= 0.04), with no other differences between weight conditions, and in the BF, EMG was 66% larger with 20FW than with 0W (*P *< 0.001), and 54% larger with 20FW than 20BW (*P *= 0.002).

The interaction of speed × weight was significant for the soleus (*F*_(1.22,12.2) _= 6.2, *P *= 0.02), LG (*F*_(2,20) _= 5.6, *P *= 0.01) and BF (*F*_(2,18) _= 4.8, *P *= 0.02). Soleus, LG and BF were significantly larger at 1.5 m/s than 1.0 m/s for all weight conditions (78–93% larger in soleus; 95–114% larger in LG; 51–81% larger in BF; *P *< 0.001 for all). Soleus EMG was ∼25% larger with 20BW than with 0W, regardless of the speed (*P *< 0.001 for both speeds); and soleus EMG was larger with 20BW than 20FW at 1.5 m/s speed only (20BW was 11% larger, *P *= 0.02). There was no difference in soleus EMG between 0W and 20FW at either speed. In the LG, EMG amplitude was larger with 20FW (0.3% at 1.0 m/s; 1% at 1.5 m/s) and 20BW (1% at 1.0 m/s; 2% at 1.5 m/s), than 0W (*P *< 0.05 for all) and was larger with 20BW than with 20FW (0.9% for 1.0 m/s and 1% for 1.5 m/s, *P *< 0.05 for both speeds). In the BF, EMG was larger during 20FW than 20BW (49% at 1.0 m/s; 72% at 1.5 m/s) and 0W (53% at 1.0 m/s; 83% at 1.5 m/s), at both speeds (*P *< 0.01 for all).

For the analysis of quadriceps activity at early stance (e.g., VL_ST_, VM_ST_, RF_ST_) and end of swing phase (e.g., VL_SW_, VM_SW_, RF_SW_), the ANOVA revealed a significant main effect of speed for both early stance and end swing phase quadriceps activity: VM_ST_ (*F*_(1,9) _= 88.7, *P *< 0.001), VL_ST_ (*F*_(1,9) _= 46.1, *P *< 0.001), RF_ST_ (*F*_(1,9) _= 28.9, *P *< 0.001), VM_SW_ (*F*_(1,7) _= 21.2, *P *= 0.002), VL_SW_ (*F*_(1,8) _= 18.2, *P *= 0.003), RF_SW_ (*F*_(1,8) _= 10.9, *P *= 0.01); indicating larger EMG amplitude with 1.5 m/s than 1.0 m/s for each of the quadriceps during each phase. A significant main effect of weight was also observed in the quadriceps activity for early stance and end of swing: VM_ST_ (*F*_(2,18) _= 34, *P *< 0.001), VL_ST_ (*F*_(2,18) _= 22.7, *P *< 0.001), RF_ST_ (*F*_(2,18) _= 21.6, *P *< 0.001), VM_SW_ (*F*_(2,14) _= 3.8, *P *= 0.048), VL_SW_ (*F*_(2,16) _= 5.0, *P *= 0.02). For early stance, *post hoc* tests revealed that the quadriceps activity was larger with 20BW and 20FW than with 0W (VL: 54–63%; VM: 50–67%; RF: 36–56%, *P *< 0.001 for all). For end swing, *post hoc* tests revealed no differences between weight conditions for the quadriceps.

### 3.2 Effects of speed and weight on joint motion

**FIGURE 3:**
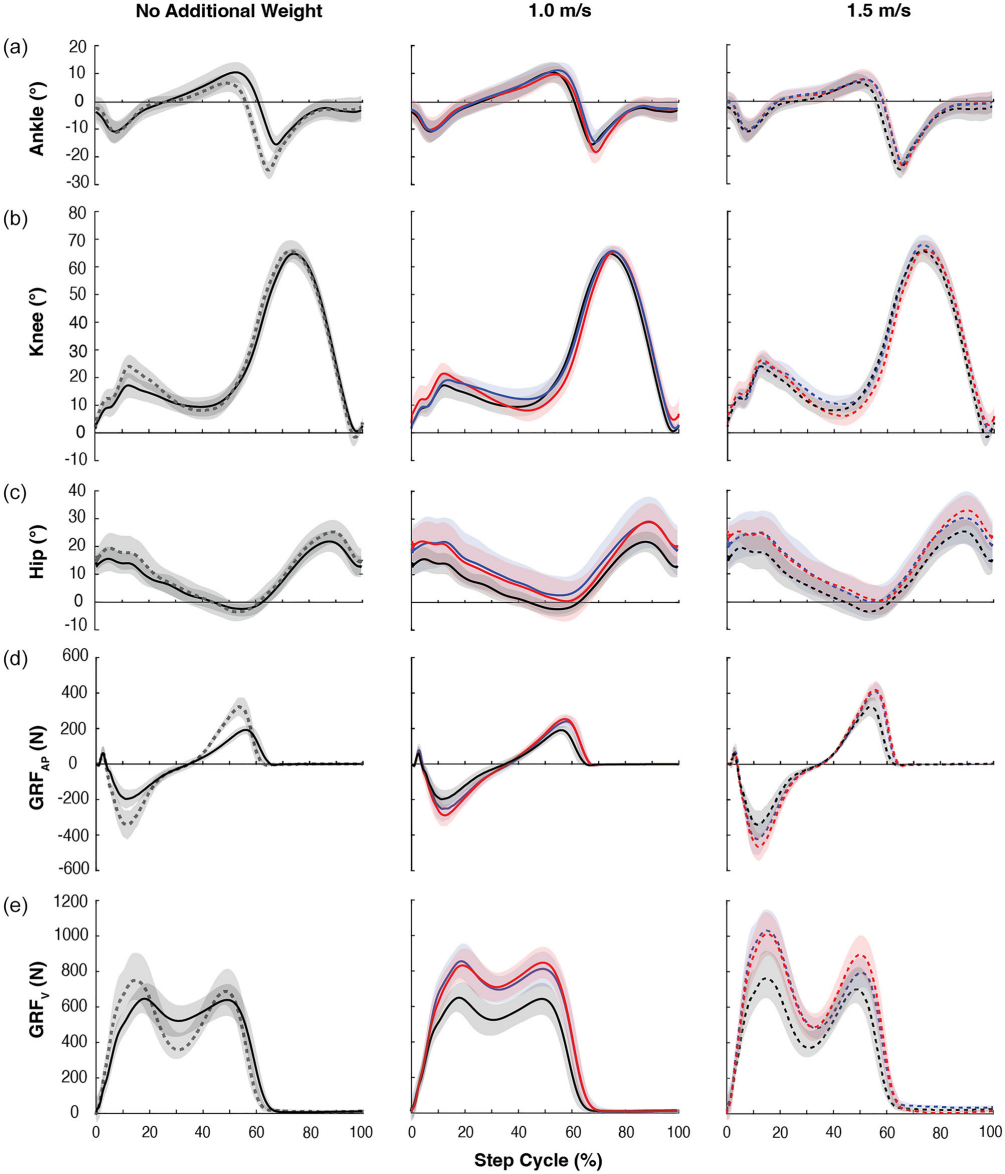
Ankle, knee and hip joint motion and GRF during walking at 1.0 m/s and 1.5 m/s with or without additional weight. Mean (±SD, indicated in shades) values across all participants are displayed for the ankle (a), knee (b), and hip (c) joint motion, and anterior–posterior GRF (GRF_AP_, d) and vertical GRF (GRF_V_, e). The leftmost column shows the no additional weight condition at 1.0 m/s and 1.5 m/s. The middle column shows all weight conditions at 1.0 m/s. The rightmost column shows all weight conditions at 1.5 m/s. For all joint motion, knee and hip flexion and ankle dorsiflexion are represented in the positive (+) direction and knee and hip extension and ankle plantarflexion are represented in the negative (−) direction. For GRF_AP_, anterior (i.e., breaking) force is represented in the negative (−) direction and posterior (i.e., propulsive) force is in the positive (+) direction. Continuous lines are for 1.0 m/s and dashed lines are for 1.5 m/s. Black lines are for no additional weight (0W), blue lines are for front weight (20FW) condition and red lines are for back weight (20BW) condition. All data are normalized to the mean step cycle duration and expressed as a percentage of the step cycle with zero as the moment of foot contact. GRF, ground reaction force.

Joint motion for ankle, knee and hip over the step cycle is shown in Figure 3 and their characteristic values are summarized in Table 1. In general, the joint range of motion was larger at 1.5 m/s than at 1.0 m/s for all three joints. With additional weight (i.e., with 20BW and 20FW), the hip joint position was in a more flexed position than with no additional weight (i.e., 0W) throughout the step cycle; this observation was consistent both at 1.0 m/s and 1.5 m/s speeds (middle and right columns, Figure 3c). In the sections below, only statistically significant effects and differences are reported.

**TABLE 1:** Summary of joint angles during walking at the two speeds and with three weight conditions.

	Median angle across the step cycle (°)		Angle at heel contact (°)		Peak flexion angle (°)		Peak extension angle (°)		Range of motion (°)	
	1.0 m/s	1.5 m/s	1.0 m/s	1.5 m/s	1.0 m/s	1.5 m/s	1.0 m/s	1.5 m/s	1.0 m/s	1.5 m/s
**Ankle**										
**0W**	−3 ± 3	−3 ± 3	−4 ± 4	−3 ± 4	11 ± 4	7 ± 3**	−6 ± 3	−25 ± 3^**^	27 ± 4	32 ± 4^**^
**20BW**	−2 ± 3**^††^**	−2 ± 2**^††^**	−3 ± 4	−1 ± 4^††**^	10 ± 4	8 ± 4^**^	−19 ± 4**^†^**	−24 ± 3^**^	29 ± 3**^†^**	32 ± 4^*^
**20FW**	−2 ± 3**^††^**	−2 ± 3**^††^**	−3 ± 4	−1 ± 4^††**^	12 ± 4	8 ± 3^†**^	−15 ± 5	−23 ± 3^††**^	27 ± 4	31 ± 3^**^
**Knee**										
**0W**	13 ± 6	16 ± 8^**^	3 ± 2	4 ± 2	65 ± 2	66 ± 4	9 ± 3	8 ± 3^*^	65 ± 2	68 ± 4^**^
**20BW**	15 ± 7	17 ± 8^**^	6 ± 5	6 ± 4	66 ± 2	66 ± 3	8 ± 4	6 ± 4^*^	63 ± 3**^††^**	65 ± 4^††**^
**20FW**	15 ± 7**^†^**	18 ± 9^†**^	3 ± 1	4 ± 2	66 ± 2	68 ± 3^††**^	12 ± 4**^††^**	10 ± 3^††*^	65 ± 3	68 ± 5^**^
**Hip**										
**0W**	9 ± 3	11 ± 5	13 ± 4	15 ± 4	22 ± 4	25 ± 4^*^	−3 ± 3	−4 ± 3	25 ± 2	29 ± 3^**^
**20BW**	13 ± 7**^†^**	16 ± 6**^†^**	20 ± 6**^††^**	23 ± 4**^††^**	29 ± 6**^††^**	33 ± 6^††*^	1 ± 7**^†^**	0 ± 5**^†^**	29 ± 3**^††^**	33 ± 5^††**^
**20FW**	15 ± 9**^†^**	17 ± 8**^†^**	19 ± 9	20 ± 9	29 ± 9	30 ± 9^*^	2 ± 7**^†^**	0 ± 6**^†^**	27 ± 4	31 ± 6^**^

*Note*: All values are reported as group mean ± SD. *n* = 11. 0W, no additional weight; 20BW, 20.4 kg of back weight carrying; 20FW, 20.4 kg of front weight carrying. Peak flexion angle: measured during mid stance for ankle, during swing for knee, and during late swing for hip. Peak extension angle: measured during terminal stance before ‘toe off’ for ankle and hip and at terminal stance before ‘heel off’ for knee. Significant differences between weight conditions (by Bonferroni as *post hoc* test) are indicated with † (*P *< 0.05) and †† (*P *< 0.01). Significant differences between speed conditions (by Bonferroni) are indicated with * (*P *< 0.05) and ** (*P *< 0.01).

#### 3.2.1 Ankle

The ANOVA revealed a significant main effect of speed on ankle range of motion (*F*_(1,10) _= 20.8, *P *< 0.001), angle at heel‐contact (*F*_(1,10) _= 27.1, *P *< 0.001), peak flexion (peak dorsiflexion, DF) (*F*_(1,10) _= 27.8, *P *< 0.001), and peak extension (peak plantarflexion, PF) (*F*_(1,10) _= 36.9, *P *< 0.001). A main effect of weight was found significant for range of motion (*F*_(2,20) _= 5.8, *P *= 0.010), median angle (*F*_(2,20) _= 20.4, *P *< 0.001), angle at heel‐contact (*F*_(2,20) _= 18.1, *P *< 0.001), peak DF (*F*_(2,20) _= 5.2, *P *= 0.02) and peak PF (*F*_(2,20) _= 12.9, *P *< 0.001). The interaction of speed × weight was significant for the range of motion (*F*_(2,20) _= 7.4, *P *= 0.004), angle at heel contact (*F*_(2,20) _= 4, *P *= 0.03), peak DF (*F*_(2,20) _= 4, *P *= 0.004) and peak PF (*F*_(2,20) _= 16.6, *P *< 0.001). *Post hoc* comparisons between speeds and weights are summarized in Table 1. The ANOVA also revealed a significant main effect of speed for the timings of peak DF and PF (*F*_(1,10) _= 43.3 and 328.5, respectively, *P *< 0.001 for both), and *post hoc* analysis indicated that both peak DF and PF occurred ∼2.8% earlier in the step cycle at 1.5 m/s than 1.0 m/s (*P *< 0.001 for both). A main effect of weight was also significant with the ANOVA for the timings of peak DF (*F*_(2,20) _= 53.3, *P *< 0.001) and peak PF (*F*_(2,20) _= 80.8, *P *< 0.001); both events occurred ∼1.75% later in the step cycle with 20BW and 20FW than 0W (*P *< 0.001 for both).

#### 3.2.2 Knee

The ANOVA revealed a significant main effect of speed on range of motion (*F*_(1,10) _= 23.7, *P *< 0.001), median angle (*F*_(1,10) _= 11.3, *P *= 0.007), angle at peak extension during late stance (*F*_(1,10) _= 4.9, *P *= 0.049) and peak flexion during early stance (*F*_(1,10) _= 64.4, *P *< 0.001). A significant main effect of weight was found on a range of motion (*F*_(2,20) _= 13.7, *P *< 0.001), median angle (*F*_(2,20) _= 4.5, *P *= 0.02), the angle at peak extension during late stance (*F*_(2,20) _= 21.6, *P *< 0.001), peak flexion during early stance (*F*_(2,20) _= 28, *P *< 0.001), heel contact (*F*_(1.16,20.2) _= 5.1, *P *= 0.04) and peak flexion during swing phase (*F*_(2,20) _= 22.5, *P *< 0.001). The interaction of speed × weight was significant for the angle at peak flexion during early stance (*F*_(2,20) _= 4.8, *P *= 0.02) and at peak flexion during swing (*F*_(2,20) _= 4, *P *= 0.03). *Post hoc* comparisons between speeds and weights are summarized in Table 1. For *post hoc* analyses of peak knee flexion during early stance (not included in Table 1), the angle was larger at 1.5 m/s than at 1.0 m/s for all weight conditions (6% larger, *P *< 0.001 for all); and between weight conditions, the angle was larger with 20BW than with 0W (5%, *P *< 0.001 and 2%, *P *= 0.001) and 20FW (2%, *P *= 0.004 and 2%, *P *= 0.02) at both 1.0 m/s and 1.5 m/s, and the angle was larger with 20FW than with 0W at 1.5 m/s only (1%, *P *= 0.03). For the analysis of event timing, the ANOVA revealed a significant main effect of speed only for peak flexion (*F*_(1,10) _= 57.8, *P *< 0.001); and *post hoc* analysis indicated that peak flexion occurred ∼1.5% earlier in the step cycle at 1.5 m/s than 1.0 m/s (*P *< 0.001). The ANOVA also found a significant main effect of weight on the timing of peak flexion (*F*_(2,20) _= 21.5, *P *< 0.001) and peak extension (*F*_(1.27,20) _= 17.6, *P *< 0.001); and *post hoc* analysis showed that peak extension occurred ∼3.5% later in the step cycle with 20BW and 20FW than with 0W (*P *< 0.01 for both), and peak flexion occurred later with 20BW than 20FW and 0W (*P *< 0.01 for both).

#### 3.2.3 Hip

The ANOVA revealed a significant main effect of speed on a range of motion (*F*_(1,10) _= 30.2, *P *< 0.001) and peak flexion angle (*F*_(1,10) _= 5.9, *P *= 0.03), and a significant main effect of weight on a range of motion (*F*_(2,20) _= 9.3, *P *= 0.001), median angle (*F*_(2,20) _= 8.2, *P *= 0.003), the angle at heel contact (*F*_(2,20) _= 8.6, *P *= 0.002), peak flexion (*F*_(2,20) _= 8.4, *P *= 0.002) and peak extension (*F*_(2,20) _= 5.6, *P *= 0.01). The *post hoc* comparisons of hip angles between speeds and weights are summarized in Table 1. For the analysis for event timing, the ANOVA indicated a significant main effect of weight for peak flexion (*F*_(2,20) _= 6.8, *P *= 0.005) and extension (*F*_(1.28,20) _= 6.1, *P *= 0.02); and *post hoc* analysis showed that peak extension occurred ∼2% later in the step cycle with 20BW than with 20FW (*P *= 0.02) and peak flexion occurred ∼1% later with 20BW than with 0W (*P *= 0.006).

### 3.3 Effects of speed and weight on temporal parameters of gait

Step cycle duration, stance phase duration, and swing phase duration for different speed and weight conditions are summarized in Table 2. In general, the step cycle duration was shorter at 1.5 m/s than at 1.0 m/s and shorter also with additional weights (i.e., 20BW and 20FW) than with no weight (i.e., 0W).

**TABLE 2:** Summary of temporal parameters during walking at two speeds and with three weight conditions.

	Step cycle duration (ms)		Stance duration (% of step cycle duration)		Swing duration (% of step cycle duration)	
	1.0 m/s	1.5 m/s	1.0 m/s	1.5 m/s	1.0 m/s	1.5 m/s
**0W**	1137 ± 47	973 ± 29^**^	63 ± 1	62 ± 2^**^	37 ± 1	38 ± 2^**^
**20BW**	1163 ± 55	974 ± 35^**^	65 ± 1^††^	63 ± 1^††**^	35 ± 1^††^	37 ± 1^††**^
**20FW**	1090 ± 72^††^	939 ± 48^†**^	65 ± 1^††^	63 ± 1^††**^	35 ± 1^††^	37 ± 1^††**^

*Note*: All values are reported as group mean ± SD. *n* = 11. 0W, no additional weight; 20BW, 20.4 kg of back weight carrying; 20FW, 20.4 kg of front weight carrying. Significant differences between weight conditions (by Bonferroni as *post hoc*) are indicated with † (*P *< 0.05) and †† (*P *< 0.01). Significant differences between speed conditions (by Bonferroni test) are indicated with* (*P *< 0.05) and ** (*P *< 0.01).

The ANOVA revealed a significant main effect of speed on the step cycle duration (*F*_(1,10) _= 360.6, *P *< 0.001;), stance phase duration (*F*_(1,10) _= 76.9, *P *< 0.001) and swing phase duration (*F*_(1,10) _= 76.9, *P *< 0.001) with *post hoc* analysis indicating a shorter step cycle (168 ms, *P *< 0.001) and stance phase (2%, *P *< 0.001), and longer swing phase (2%, *P *< 0.001) at 1.5 m/s than at 1.0 m/s. A significant main effect of weight was also found for the step cycle duration (*F*_(2,20) _= 20.2, *P *< 0.001), stance phase duration (*F*_(2,20) _= 29.2, *P *< 0.001) and swing phase duration (*F*_(2,20) _= 29.2, *P *< 0.001); and *post hoc* analysis indicated a shorter step cycle duration at 20FW compared to 0W (40 ms, *P *= 0.006) and 20BW (54 ms, *P *< 0.001); and longer stance duration and shorter swing duration with 20BW (2%) and 20FW (2%) than with 0W (*P *< 0.01 for both). A speed × weight interaction was significant only for the step cycle duration (*F*_(2,20) _= 6.9, *P *= 0.005), with *post hoc* analyses indicating a shorter step cycle duration at 1.5 m/s than 1.0 m/s for all weight conditions (150–189 ms range, *P *< 0.001 for all), and a shorter step cycle duration with 20FW than with 20BW (73 ms at 1.0 m/s; 35 ms at 1.5 m/s, *P *< 0.001) and with 0W (47 ms at 1.0 m/s, *P *= 0.008 and 34 ms at 1.5 m/s, *P *= 0.03).

### 3.4 Effects of speed and weight on the soleus and LG *H*‐reflexes

**FIGURE 4:**
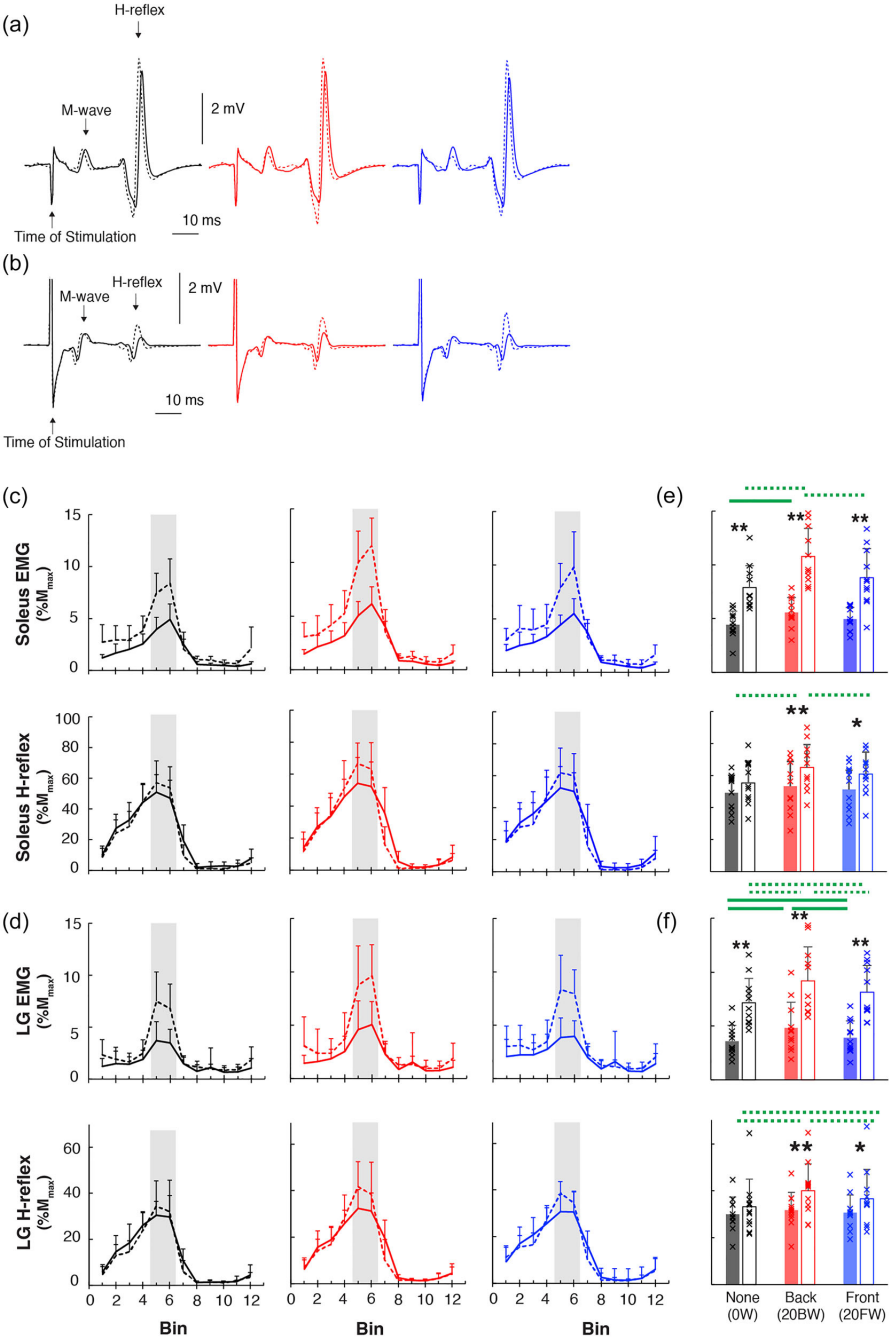
Mid–late stance soleus and LG muscle activity and H‐reflexes. (a, b) Examples of peristimulus EMG sweeps for the soleus (a) and LG (b) in mid–late stance for each speed and weight condition from a representative participant. (c, d) Mean EMG and H‐reflex amplitudes (+SD) for soleus (c) and LG (d) across all participants over the step cycle, which was divided into 12 bins of equal duration. H‐reflex and EMG amplitudes are expressed as a percentage of *M*_max_ values measured during standing. The line type and colour represent each speed and weight conditions (continuous, 1.0 m/s; dashed, 1.5 m/s; black, no additional weight (0W); blue, front weight (20FW); red, back weight (20BW)). (e, f) Bar plots representing the mean EMG burst amplitudes (e) and H‐reflex amplitudes (f) during mid–late stance (highlighted in grey in c, d). Filled bars are for 1.0 m/s and open bars are for 1.5 m/s. Each individual participant's value is displayed with ‘×’ in each panel for each condition. Asterisks denote differences between 1.0 m/s and 1.5 m/s for each weight condition (* *P *< 0.05, ** *P *< 0.01, by paired comparison with Bonferroni correction). Green lines above bar plots indicate significant differences between the weight conditions: continuous lines for 1.0 m/s and dotted lines for 1.5 m/s (*P *< 0.05, by Bonferroni test). EMG, electromyography; LG, lateral gastrocnemius; *M*_max_, maximum M‐wave.

Figure 4 shows the soleus and LG H‐reflexes in the mid–late stance of walking (Figure 4a, b) and across the step cycle, together with the locomotor EMG (Figure 4c, d, all values are shown as group mean + SD) for all six speeds and weight conditions. Phase‐dependent modulation of H‐reflex did not appear to differ across different speed and weight conditions. Mid–late stance H‐reflexes were larger at 1.5 m/s than at 1.0 m/s only in added weight conditions, but this increase was not as consistent or striking as the amplitudes of corresponding EMG (Figure 4c, d). H‐reflexes of 20BW and 20FW conditions were larger than those of 0W only during the 1.5 m/s speed.

The soleus H‐reflex modulation indices over the step cycle were 96.5–98.6 across six different speed and weight conditions, and there were no significant effects of speed or weight: *F*_(1,10) _= 3.8, *P *= 0.08 for speed and *F*_(1.26,12.6) _= 0.7, *P *= 0.52 for weight by ANOVA. The LG H‐reflex modulation indices were 96.4–97.2 across six different conditions, and there were no significant effects of speed or weight: *F*_(1,10) _= 2.7, *P *= 0.13 for speed and *F*_(1.24,12.4) _= 0.2, *P *= 0.83 for weight. The bins with the highest H‐reflex amplitude also did not differ across six different conditions (bin 5 or 6 for all).

For the mid–late stance phase H‐reflexes, the ANOVA revealed significant main effects of speed and weight for the soleus H‐reflex (*F*_(1,10) _= 7.8, *P *= 0.02 for speed; *F*_(2,20) _= 12.6, *P *< 0.001 for weight) and LG H‐reflex (*F*_(1,10) _= 5.7, *P *= 0.04 for speed; *F*_(2,20) _= 13.9, *P *< 0.001 for weight). The speed × weight interaction was significant for both the soleus (*F*_(2,20) _= 3.9, *P *= 0.04) and LG H‐reflexes (*F*_(2,20) _= 6.1, *P *= 0.009). In the *post hoc* comparisons, the soleus and LG H‐reflexes were larger at 1.5 m/s than 1.0 m/s only when bearing additional weight (i.e., 20BW (8%, *P *= 0.01 for LG and 12%, *P *= 0.008 for soleus) and 20FW (6%, *P *= 0.04 for LG and 8%, *P *= 0.02 for soleus)). For the soleus H‐reflex, the amplitude was larger with 20BW than with 0W (10%, *P *< 0.001) and 20FW (6%, *P *= 0.03) at 1.5 m/s only. For the LG H‐reflex, the amplitude was larger with 20FW (3%, *P *= 0.01) and 20BW (7%, *P *= 0.001) than with 0W, and 20BW was larger than that with 20FW (3%, *P *= 0.04) at 1.5 m/s.

### 3.5 Effects of speed and weight on ankle rotational speed, ankle displacement, and rate of GRF development in mid–late stance

**FIGURE 5:**
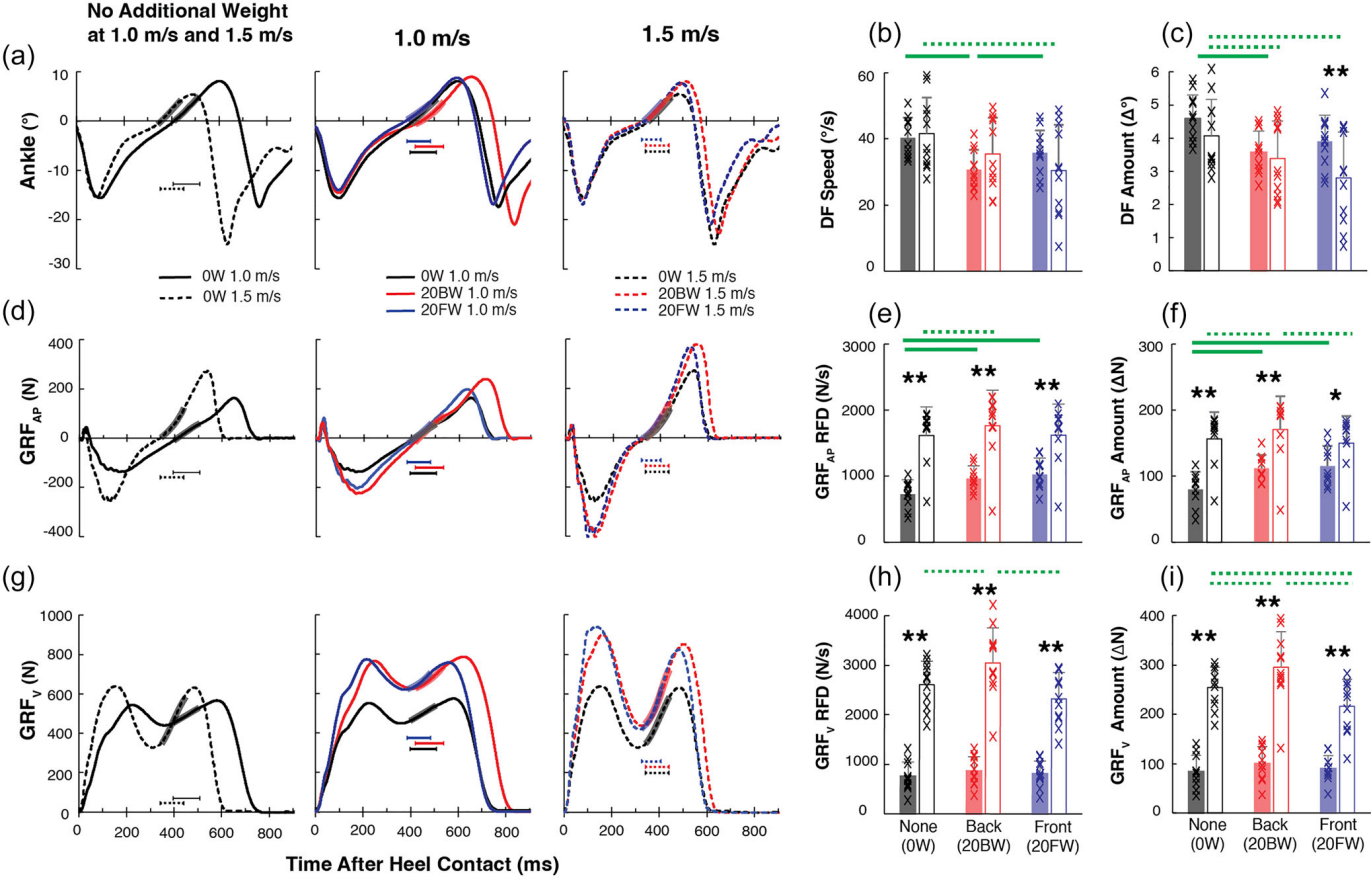
Ankle rotational speed, GRF and RFD during mid–late stance. Example traces of locomotor ankle joint motion (a), GRF_AP_ (d), and GRF_V_ (g) from a representative participant. The leftmost column shows 1.0 m/s and 1.5 m/s conditions with no additional weight. The middle column shows all weight conditions at 1.0 m/s. The rightmost column shows all weight conditions at 1.5 m/s. Continuous lines are for 1.0 m/s and dashed lines are for 1.5 m/s. Black lines are for no additional weight (0W), blue lines are for front weight (20FW), and red lines are for back weight (20BW) conditions. The highlighted parts of the traces indicate the time periods of the data used to calculate the amount and speed of change in ankle rotation (b, c), GRF_AP_ (e, f), and GRF_V_ (h, i) over mid–late stance (35–45% of the step cycle). The time periods are also indicated with horizontal lines below the signal traces. Bar plots represent the average DF rotational speed (b) and amount of change (c), GRF RFD (e, h) and GRF amount of change (f, i). Filled bars are for 1.0 m/s and open bars are for 1.5 m/s. Each ‘×’ symbol represents each individual participant's value. Asterisks denote differences between 1.0 m/s and 1.5 m/s for each weight condition (* *P *< 0.05 and ** *P *< 0.01, by Bonferroni test). Green lines above bar plots indicate significant differences between the weight conditions: continuous lines for 1.0 m/s and dotted lines for 1.5 m/s (*P *< 0.05, by Bonferroni test). DF, dorsiflexion; GRF, ground reaction force; GRF_AP_, anterior‐posterior GRF; GRF_V_, vertical ground reaction force; RFD, rate of force development.

Ankle joint motion and GRFs across the step cycle (group mean + SD) are shown in Figure 3, and typical examples of ankle joint motion and GRFs over the stance phase from a representative individual are displayed in Figure 5a, d, g. The amount and rate of change in ankle dorsiflexion and GRF (group mean+SD) during the mid–late stance phase are shown in Figure 5b, c, e, f, h, and i. Generally, the ankle dorsiflexion amount during mid–late stance was less at 1.5 m/s than at 1.0 m/s while the rotational speed was maintained between the two speeds (Figure 5 a–c). GRF_AP_ and GRF_V_ changed more and faster at 1.5 m/s than at 1.0 m/s (Figure 5d–i). The amount and speed of dorsiflexion were less with 20FW or 20BW than with 0W. The amount and rate of GRF development was larger with 20BW than with 0W for both GRF_AP_ and GRF_V_ but was only larger for GRF_V_ during 1.5 m/s.

#### 3.5.1 Ankle dorsiflexion

The ANOVA revealed significant main effects of speed and weight on ankle dorsiflexion amount (*F*_(1,10) _= 5.8, *P = *0.04 for speed; *F*_(2,20) _= 13.5, *P *< 0.001 for weight) as well as a significant interaction between them (*F*_(2,20) _= 8.9, *P *= 0.002). *Post hoc* comparisons revealed that ankle dorsiflexion amount was significantly larger during 1.0 m/s than at 1.5 m/s during 20FW only (0.6° larger, *P *= 0.002); no other speed differences were observed. Between weight conditions, ankle dorsiflexion amount was larger at 0W than 20BW at both 1.0 m/s and 1.5 m/s (1° larger, *P *< 0.001 and *P *= 0.03), and larger with 0W than with 20FW at 1.5 m/s (1.2° larger, *P *< 0.001). For ankle rotational speed, the ANOVA indicated a significant main effect of weight (*F*_(2,20) _= 9.4, *P *= 0.001) and interaction between speed and weight (*F*_(2,20) _= 9.4, *P *= 0.001). *Post hoc* comparisons revealed that ankle rotational speed was slower with 20BW than with 0W (10°/s slower, *P *= 0.001) and 20FW (5°/s slower, *P *= 0.02) at 1.0 m/s, but at 1.5 m/s, ankle rotational speed was slower with 20FW than with 0W (11°/s slower, *P *= 0.002).

#### 3.5.2 GRF_AP_

The ANOVA revealed significant main effects of speed (*F*_(1,8) _= 26, *P *< 0.001) and weight (*F*_(2,16) _= 20.5, *P *< 0.001) and their interaction (*F*_(2,16) _= 11.5, *P *< 0.001) on GRF_AP_ amount. *Post hoc* comparisons revealed that GRF_AP_ amount was larger at 1.5 m/s compared to 1.0 m/s for all weight conditions (76 N, *P *< 0.001 for 0W, 59 N, *P *= 0.003 for 20BW, 35 N, *P *= 0.02 for 20FW). Between weight conditions, GRF_AP_ amount was larger with 20BW than with 0W at both 1.0 m/s and 1.5 m/s (32 N, *P *< 0.001 and 14 N, *P *= 0.04, respectively), and larger than 20FW at 1.5 m/s only (21 N, *P *= 0.02). 20FW was larger than 0W at 1.0 m/s only (35 N, *P *= 0.003). For the RFD, ANOVA indicated significant main effects of speed and weight (*F*_(1,8) _= 46.5, *P *< 0.001 for speed; *F*_(2,16) _= 24.3, *P *< 0.001 for weight) as well as a significant interaction between them (*F*_(2,16) _= 14.1, *P *< 0.001). *Post hoc* analyses revealed that RFD for GRF_AP_ was larger at 1.5 m/s compared to 1.0 m/s for all weight conditions (594–888 N/s range, *P *< 0.001 for all). Between weights, RFD was larger with 20BW than 0W at both 1.0 m/s (238 N/s, *P *< 0.001) and 1.5 m/s (148 N/s, *P *= 0.04), and larger with 20FW than with 0W at 1.0 m/s (298 N/s, *P *< 0.001).

#### 3.5.3 GRF_V_

The ANOVA revealed significant main effects of speed (*F*_(1,10) _= 173, *P *< 0.001) and weight (*F*_(2,20) _= 11.1, *P *< 0.001) and their interaction (*F*_(2,20) _= 13.4, *P *< 0.001) on GRF_V_ amount. *Post hoc* comparisons revealed that the GRF_V_ amount was larger at 1.5 m/s than at 1.0 m/s for all weight conditions (126–195 N range, *P *< 0.001 for all). Between weight conditions, the GRF_V_ amount was larger with 20BW than with 20FW (79 N, *P *= 0.002) and 0W (41 N, *P *= 0.03) at 1.5 m/s, and larger with 0W than with 20FW at 1.5 m/s (38 N, *P *= 0.05). For RFD of GRF_V_, ANOVA indicated significant main effects of speed and weight (*F*_(1,10) _= 233.4, *P *< 0.001 for speed; *F*_(2,20) _= 9.9, *P *< 0.001 for weight) as well as a significant interaction (*F*_(2,20) _= 11.5, *P *< 0.001). *Post hoc* analyses revealed that RFD for GRF_V_ was larger at 1.5 m/s than at 1.0 m/s for all weight conditions (1516–2182 N range, *P *< 0.001 for all); and between weight conditions, was larger with 20BW than with 0W (440 N, *P *= 0.009) and 20FW (722 N, *P *= .004) at 1.5 m/s.

#### 3.5.4 Propulsive force generation in GRF_AP_

The ANOVA indicated significant main effects of speed (*F*_(1,8) _= 50.14, *P *< 0.001) and weight (*F*_(2,16) _= 59.62, *P *< 0.001) on GRF_AP_ impulse calculated over the entire propulsive phase. *Post hoc* analyses revealed that propulsive impulse was larger at 1.5 m/s than at 1.0 m/s (9 N s, *P *< 0.001). Between weight conditions, the propulsive impulse was larger with 20BW and 20FW than with 0W (15 N s for 20BW and 9 N s for 20FW, *P *< 0.001 for both), and larger with 20BW than 20FW (5 N s, *P *= 0.03).

## 4 DISCUSSION

In this study, we sought to examine how human adults manage commonly encountered additional demands during walking: going faster and carrying additional weight, through the measurements of EMG, H‐reflexes, joint motion and GRF. We found that faster walking was accompanied by a universal increase in burst EMG amplitude across flexors and extensors of upper and lower leg muscles (although the plantarflexor activity increase was most notable) while maintaining their burst patterns of activity; we also observed an increased range of motion at the ankle, knee and hip joints, and shortened step cycle duration. In bearing additional weight, we found consistent increases in the extensor (especially the quadriceps) activity and a shift in a hip angle toward a more flexed position across the step cycle. When the participants were tasked to walk faster and carry additional weight at the same time, changes in locomotor EMG and joint motion concerning the knee and hip joints displayed added‐up features of those two additional demands; in contrast, changes in the plantarflexor activity and ankle joint motion appeared to be more complex. Below, we discuss these EMG and joint motion features of walking faster and carrying additional weight and their potential mechanisms.

### 4.1 Increased EMG activity across multiple flexors and extensors when walking fast

In this study, we observed that faster walking was accompanied by increased burst EMG amplitude across flexors and extensor muscles in both the upper and lower leg while maintaining their burst patterns of activity (Figure 2). Considering the phasic nature of locomotor EMG activity, it makes sense that such EMG amplitude increases across multiple muscles would lead to the joints moving more robustly through their pattered cycles (see Figure 3 and Table 1). Then, the question is where this global increase of locomotor EMG activity would come from. Because the EMG increase was not limited to specific muscle groups but clearly observable across flexors and extensors in upper and lower portions of the leg, a plausible explanation is that it is from an increase in supraspinal descending drive to the network of spinal neurons that is capable of generating patterned motor output (known as the central pattern generator, CPG; Grillner & Kozlov, 2021; Rossignol et al., 2006; Zehr, 2005). This possibility is supported by Dimitrijevic et al. (1998), in which increasing the intensity of spinal cord stimulation (which could be viewed as pseudo‐supraspinal drive to the CPG) led to an increase in EMG amplitude across multiple flexors and extensors in the leg of an individual with chronic complete spinal cord injury. Dimitrijevic et al. (1998) also observed shortening of EMG burst cycle duration with increasing spinal stimulation intensity; this would align well with the present observation of shortened step cycle duration during faster walking (see Table 2). Overall, together with the previous human and animal studies (Brocard & Dubuc, 2003; Caggiano et al., 2018; Grillner & Kozlov, 2021), the present data support that walking faster comes with increased descending drive to the locomotor CPG (or CPG‐like spinal neuronal network), at least partly.

### 4.2 Increased plantarflexor activity during faster walking

When walking faster, the EMG burst amplitude increases universally across flexors and extensors; at the same time the extent of EMG increase can be most noticeable in the ankle plantarflexors, soleus and LG (as seen in Figure 2). This is not necessarily a surprise since the plantarflexors are the actively contracting muscles during mid–late stance and primary contributors to propulsive force generation (Griffin et al., 2003; Kulmala et al., 2016; Neptune et al., 2001, 2008). As to what drives plantarflexors during walking, along with the descending input, it is thought to be the muscle afferent input (af Klint, Cronin, et al., 2010; Grey et al., 2004, 2007; Mazzaro et al., 2005; Sinkjaer et al., 2000; Stephens & Yang, 1999). Thus, it is possible that the same sensory afferents would contribute to increasing plantarflexor activity during mid–late stance (i.e., push‐off) when walking faster. If so, which afferent and its pathway would be enhancing the plantarflexor burst during faster walking?

In this study, when participants were walking faster, we observed the modest extent of H‐reflex amplitude increase in mid–late stance (by +12–18%), which did not match the large amount of EMG increase that occurred during this phase (by +100% for soleus and +133% for LG, Figure 4c). Furthermore, ankle DF speed, which would serve as an approximation of the rate of muscle length change, was not different between the two walking speeds (Figure 5b). Thus, the present data do not support the possibility of Ia afferents and/or their excitatory pathways as primary contributors to the enhanced plantarflexor burst during faster walking. Previously, Cronin et al. (2009) measured soleus stretch reflexes and fascicle length change during three different speeds of walking and found no difference in stretch reflex amplitude across three different walking speeds while the amount of fascicle length change was less at a faster walking speed. Altogether, the available findings to date are in line with past studies that led to the view that Ia afferents are not primary contributors to the soleus EMG generation during the propulsive phase of walking (e.g., Sinkjær et al., 2000).

To consider the potential involvement of the group II pathway in the plantarflexor burst during the mid–late stance phase, we measured ankle displacement, which would be linked to muscle length change. We did not find a significant difference in ankle displacement over mid‐stance between the two walking speeds (Figure 5c). This may suggest that it is improbable that the length‐sensitive group II afferents contribute to enhancing the plantarflexor burst when walking faster.

Next, to consider the group Ib pathway's involvement, as approximations of muscle loading in the triceps during the mid–late stance phase, we calculated the amount and rate of GRF development; Ib afferents are known to fire in response to changes in loading amount and loading rate (Appenteng & Prochazka, 1984; Davies et al., 1995; Donelan et al., 2009; Houk & Henneman, 1967). When walking faster, the amount of GRF_V_ increase in the mid–late stance phase was 170 N (203%) more and its RFD was 1851 N/s (244%) more than those during walking near self‐selected speed (i.e., 1.0 m/s). Similarly, the amount of GRF_AP_ increase in the mid–late stance phase was 76 N (94%) larger and its RFD was 888 N/s (122%) larger during faster walking (see Figure 5d–i). Increases in these force measures were so large that we could be certain of increased Ib firing during faster walking. Thus, these results would further support the possibility that Ib afferents contribute to increasing plantarflexor EMG burst amplitude in mid–late stance when walking faster.

### 4.3 On potential neural mechanisms of walking fast

All in all, an emerging picture on walking fast is that both supraspinal and spinal mechanisms are involved. When the human participant was asked to walk at a distinctively faster (i.e., +50%) speed in this study, there was a universal increase in EMG activity (Figure 2) and acceleration of the step cycle (Table 2), as opposed to, for example, an increase in activity of a specific group of muscles or changes in stance/swing phase balance, suggesting that a change was brought in upstream of the spinal neural circuit that excites multiple motoneuron pools in coordination (i.e., locomotor CPG or CPG‐like network). Alongside the influence from supraspinal drive, motoneurons involved in locomotion also receive excitatory and inhibitory input from sensory afferents directly, such as from Ias (Capaday & Stein, 1986, 1987a; Edamura et al., 1991; Mrachacz‐Kersting et al., 2004; Nielsen & Sinkjaer, 2002b; Yang et al., 1991; Yavuz et al., 2014), or through interneuronal pathways (Kido et al., 2004b; Nakajima et al., 2014; Stephens & Yang, 1996; Zehr et al., 1998). In the present study, when a person walked faster, a large increase in the mid–late stance plantarflexor activity (Figure 2) was accompanied with large increases in GRF measures (Figure 5), which would suggest increased Ib firing (see discussion in the section above) that could enhance the triceps surae motoneuron firing (Donelan & Pearson, 2004a; Donelan et al., 2009). While we could not be certain which comes first – the increased plantarflexor activity increased force generation (i.e., increased vertical and anterior‐posterior GRF) or the increased force feedback increased plantarflexor activity – it would be reasonable to assume that sensory feedback from Ib afferents helps to reinforce and ensure plantarflexor activity for generating propulsive force to produce an intended speed.

The idea of CPG expression being shaped or reinforced by sensory input has been well described. Sensory input from proprioceptive afferents is suggested to be essential for setting the timing and magnitude of the cyclic EMG activity (Conway et al., 1987; Donelan & Pearson, 2004b; Grillner, 2021; Hultborn & Nielsen, 2007; Hultborn et al., 1998; Nielsen & Sinkjaer, 2002a) and shaping locomotor behaviors (Lam & Pearson, 2002; Rossignol et al., 2006). For example, vibration to activate muscle spindle afferents in the hip flexors reduces the duration of extensor activity in stance phase (Hiebert et al., 1996), whereas stimulating the group I afferents in extensor muscles prolongs the stance phase (Whelan et al., 1995). Loading of the limbs (presumably enhancing Ib firing) prolongs the stance phase (Pang & Yang, 2000), while unloading shortens it (Gorassini et al., 1994). In sum, the available pieces of evidence (including the findings from this study) suggest that we walk faster by increasing supraspinal drive to the locomotor CPG (or CPG‐like network) while utilizing the sensory afferent feedback to produce propulsive force generating EMG activity to meet the task demand of increasing speed. The fast‐conducting Ia afferents likely play an important role if the walk is perturbed unexpectedly; experimentally, they have been shown to play an important role in generating a corrective response in such situations (Mazzaro et al., 2006, 2005; Sinkjaer et al., 1996).

### 4.4 Increased extensor EMG activity when carrying additional weight

In this study, we observed that walking with additional weight was accompanied by increases in the activity of upper and lower leg extensor muscles (see Figure 2); the quadriceps EMG (VM, VL, RF) over the end of swing to early stance was 31–56% larger and the soleus and LG EMG in mid–late stance was 25% larger with additional weight at 1.0 m/s. Different from walking faster, carrying additional weight did not appear to increase EMG activity universally across multiple muscles. For instance, the activity of the dorsiflexor TA was barely larger by ∼10% with 20BW; the functional significance of such a limited change is questionable (see Figure 2). BF EMG increase was only observed with 20FW and not 20BW, which is potentially attributable to external rotation of the leg that was not captured with the present measures. Functionally it makes sense that the activity of leg extensors becomes larger when carrying additional weight, as they are anti‐gravity muscles (Winter, 1983a, 1983b; Neptune et al., 2001; Pandy & Andriacchi, 2010; Sloot & van der Krogt, 2018) that help maintain an upright posture during walking. Stephens & Yang (1999) also reported somewhat similar increases in the activity of extensors (+78% in VL and +14% in soleus) when carrying an additional 30% of bodyweight (Stephens & Yang, 1999). Where could this increase in extensor EMG activity come from? Because it was limited mostly to the extensors, it would not be from a general increase in descending drive to the spinal network that involves both the flexors and extensors (i.e., CPG). Rather, it could be from the increased afferent input that primarily affects extensors. Pang & Yang (2000) observed prolongation of stance phase with increasing loading in infants, in whom supraspinal descending drive is not fully available yet. Prolongation of stance phase duration with added loads has also been reported in adults (McGowan et al., 2008). The implication was that afferent input from load‐sensitive receptors prolonged excitation of extensors and thereby stance phase (Pang & Yang, 2000; Whelan et al., 1995). Indeed, this aligns well with the present observation of slight yet consistent effects of additional weight on stance duration (see Table 2). Overall, together with the previous human and animal studies (Conway et al., 1987; Donelan & Pearson, 2004a; Donelan et al., 2009; Gossard et al., 1994; Grey et al., 2002), the present data support the view that increased extensor activity with additional weight likely comes from increased load‐sensitive afferent input to the extensor motoneuron pools, at least partly.

### 4.5 Increased quadriceps activity when carrying additional weight

Similar to reports by Stephens & Yang (1999) and Lindner et al. (2012), in which the quadriceps showed disproportionally larger EMG activity increases than the plantarflexors when the participants had to bear additional weight, we saw more noticeable increases in the quadriceps EMG activity with additional weight in the present study (Figure 2) (Lindner et al., 2012; Stephens & Yang, 1999). During walking (without additional weight), quadriceps EMG activity peaks at two connecting yet distinct phases of the step cycle: end swing and early stance. In end swing, the limb is unloaded (i.e., off the ground), the knee nears its full extension, and the hip starts to extend after reaching its peak flexion (see Figure 3). Thus, the quadriceps activity in this phase would be mostly for stabilizing the knee (with VM and VL) and hip (with RF) in preparation for upcoming heel‐contact and would be unlikely to require robust input from homonymous muscle afferents. Even if the knee joint motion is perturbed by several degrees, it results in little excitation of the quadriceps in this phase. Mrachacz‐Kersting et al. (2004) found limited stretch reflex activity (presumably Ia, II and Ib origin) in the quadriceps in late swing and brisk quadriceps stretch reflexes of multiple latency components (i.e., Ia, II and Ib origin) in early stance, indicating the availability of muscle afferent pathways for enhancing EMG activity during early stance but not late swing (Mrachacz‐Kersting et al., 2004). In early stance, while joint stabilization continues to be critical, the limb becomes loaded, and the knee goes through flexion (Figure 3). Under this condition, quadriceps muscle afferents could be firing robustly (Donelan & Pearson, 2004a; Donelan et al., 2009) and contributing to EMG activity. Given the large increase in GRF that occurs in early stance when carrying additional weight (see Figures 3 and 5), excitatory pathways of load sensitive Ib afferent origin could be contributing to the early stance quadriceps EMG increase in this condition.

In the present study, when we looked at the quadriceps activity in the end swing and early stance separately, the effect of carrying additional weight was very clear in early stance but not in end swing (Figure 2). Together with very limited changes in the flexor activity, this phase specific increase in the quadriceps activity suggests that carrying additional weight during walking is unlikely to involve changes in general descending drive to the CPG or CPG‐like network in the spinal cord. Rather, the current findings may support the possibility that necessary changes in extensor activity to meet the increase task demand of carrying additional weight are generated largely through afferent pathways.

### 4.6 Increased hip flexion when carrying additional weight

One of the notable effects of carrying additional weight was that it affected the hip joint position across the step cycle (+4‐6°), keeping the hip more flexed regardless of the position of the weight (Table 1, Figure 3c). A previous study of 10% and 15% bodyweight loading (Fiolkowski et al., 2006) found increased forward trunk lean, increased hip flexion and decreased hip extension with backpack loading and the inverse effects with front pack loading, suggesting a possible link between hip flexion and trunk flexion. Intuitively, one might assume that the person would adjust the trunk position in compensation to the position of the added weight to maintain the body's centre of mass. However, potential pelvic tilt in these weight carrying conditions could lead to co‐occurrence of trunk extension and hip flexion. Since the present study did not capture sagittal plane trunk motion, it remains unclear whether the observed hip flexion was due to increased trunk flexion.

### 4.7 How do we walk faster and carry additional weight at the same time?

As discussed above, it appears that we walk faster by increasing supraspinal descending drive to the locomotor CPG while utilizing the muscle afferent feedback (e.g., Ib) to generate the plantarflexor EMG activity sufficient to produce the desired amount of propulsive force; and we walk with additional weight by increasing the leg extensor activity through the afferent drive. Now, how do we do both?

As we look at the quadriceps activity (Figure 2), the distinct impacts of walking faster and bearing additional weight on the end swing and early stance EMG are quite recognizable; each task demand imposed its own effect on the quadriceps EMG and when the two demands were brought in together, their individual effects were added up in the EMG amplitude. For example, the VM activity in the early stance was larger by 88% when walking faster (at 1.5 m/s, with no additional weight) and larger by +48–67% when carrying additional weight, compared to the condition of 1.0 m/s with no additional weight; the activity close to their sum was what was observed when the participants walked faster (at 1.5 m/s) with additional weight (i.e., +158–177%). Such “add‐up” effects appear to exist similarly for the TA and BF. A similar picture emerges from the hip joint motion data; for example, the increased range of motion that occurs when walking faster remains present when the additional weight was added and the increases observed in the median hip angle across the step cycle with additional weight at 1.0 m/s walking speed was very similar to that observed at 1.5 m/s walking speed (see Table 1 and Figure 3). Together, the present findings support the possibility that the mechanisms associated with walking faster and bearing additional weight co‐exist in parallel to regulate and modulate locomotor EMG activity that involves knee and hip joint motion.

When we look at the ankle plantarflexor activity and ankle joint motion, the effects of walking faster and bearing additional weight did not simply add up. First, the demand for walking faster was accommodated by clear and large increases in the mid–late stance soleus and LG activity (i.e., +100% for soleus and +133% for LG). In contrast, the demand of bearing additional weight produced statistically significant but not too robust effects at 1.0 m/s (e.g., +26% with 20BW and +12% with 20FW for soleus). Yet, at 1.5 m/s, the effects of bearing additional weight appear more clearly; in the soleus, the mid–late stance activity was increased by +143% with 20BW and +98% with 20FW, for example. The increased plantarflexor activity in mid–late stance was somewhat expected because both walking faster and bearing additional weight would demand more propulsive force generation (Kulmala et al., 2016; Neptune et al., 2001, 2008). What was not expected was a relatively limited effect of additional weight on plantarflexor activity at 1.0 m/s. At 1.0 m/s, additional weight also had little effect on approximations of muscle afferent excitation and/or their related pathways that are thought to contribute to locomotor plantarflexor activity in general (af Klint et al., 2010a; Grey et al., 2004, 2007; Mazzaro et al., 2005; Sinkjaer et al., 2000; Stephens & Yang, 1999); some of the GRF measures (approximations of the triceps loading, Ib excitatory mechanisms) and the soleus and LG H‐reflexes (measures of the excitability of the Ia excitatory pathway) showed no effects of additional weight at 1.0 m/s. At 1.5 m/s, the GRF measures and the soleus and LG H‐reflexes showed the effects of additional weight, partially explaining where the increased plantarflexor activity in mid–late stance may come from. Overall, for the generation and modulation of plantarflexor activity and resulting ankle joint motion, the mechanisms associated with walking faster and bearing additional weight are not easily separable; they likely interact and/or overlap to some extent. For instance, the load‐sensitive Ib afferents would increase firing in response to increases in loading amount (e.g., 0W vs. 20BW or 20FW in Figure 5g and i) or loading amount itself and increases in loading rate (e.g., 1.0 m/s vs. 1.5 m/s in Figure 5g and h) (Davies et al., 1995; Houk & Henneman, 1967). Thus, Ib afferents could be contributing to enhanced excitation of plantarflexor motoneurons in both walking faster and carrying additional weight. On the other hand, the contribution of muscle spindle afferents to the plantarflexor activity when those additional demands are brought in is much less clear. For the Ia contribution, although the H‐reflex amplitude does not increase as much as the ongoing (i.e., background) EMG amplitude (Figure 4), the fact that we could elicit the H‐reflexes in mid–late stance and that the H‐reflexes were larger when bearing additional weight at 1.5 m/s suggest that the Ia excitatory pathways are available when the task demands increase. At the same time, the fact that the ankle dorsiflexion in mid–late stance tends to decrease (in both the amount and the rate) with additional weight (Figure 5a–c) poses a question of whether these spindle afferent pathways are used at all to increase plantarflexor activity even if the pathways themselves remain accessible. However, despite these facts, we recognize that alternative interpretations remain possible.

### 4.8 Methodological limitations and neurophysiological considerations

In this study, to implement different speed and weight conditions in one experiment while maintaining the EMG recording condition and minimizing participant fatigue (both physical and mental), we had to use a fixed order of six different walking conditions (see Figure 1). By keeping the experimental duration as short as possible, the present procedures produced little to no fatigue and deterioration of EMG recording (see Section 2.6), as we hoped. To reduce the concern of a fixed experimental order affecting the data, before this study, we tested the same walking conditions in different orders in several different individuals as preliminary experiments. In those preliminary datasets, the observations were very similar to those of this study. Thus, we are confident that the fixed order of conditions used in this study would have a limited impact on the presently obtained data.

As an experimental set‐up to study the effects of additional weight, adding weight near the body's centre of mass would have caused less disturbance to the upright posture. In the present study, we tried to emulate carrying a backpack or carrying a heavy box in front, to examine how we carry additional weight in our everyday life. For good or bad, these seemed to produce mixed effects of bearing additional weight and weight‐position‐dependent postural adjustment in this study. For example, the distinct effect of the front weight (20FW) condition was unique to BF, as it was likely for producing external rotation at the knee and hip (Figure 3d) not for bearing weight itself (as the back weight condition did not produce similar effects). At the same time, the effects of additional weight appear consistently across the quadriceps (Figure 2e–g) across front and back weight conditions. Here, we acknowledge the potential unique effects that the present weight‐carrying set‐ups might have caused on the obtained data.

In the present study, to control and impose two task demands at once and to measure GRFs, we made our participants walk on an instrumented treadmill. Since EMG and joint kinematics could differ between overground and treadmill walking to some extent (Lee & Hidler, 2008; Ochoa et al., 2017), the present findings may not be immediately transferable to overground walking. Further, in real‐life overground walking, people would not walk fast when carrying an additional weight of a significant amount. If and to what extent the spinal and supraspinal mechanisms suggested in the present study are involved when people walk faster and with additional weight overground are yet to be determined.

In interpreting the reflex amplitude of the muscle in motion, we need to be cognizant of the excitability of motoneuron pools at the time of reflex elicitation. In the present study, we found that the mid–late stance H‐reflex was larger when walking faster; however, this does not immediately mean a higher excitability of the H‐reflex pathway. Because the H‐reflex amplitude increases with increasing level of motoneuron pool excitability (which is reflected in background EMG) (Capaday & Stein, 1987a, 1987b), H‐reflexes could be larger just because the excitability of the motoneuron pool is higher (i.e., background EMG is higher) not because the reflex gain is higher. Thus, the fact that during faster walking the increases in soleus and LG EMG was much more robust than those in the H‐reflexes (Figure 4) in the present study would indicate, if anything, that the reflex gain was lower when walking faster.

When examining EMG activity of sensory afferent origins, one must consider the dynamic nature of the relationship between muscle activity and joint rotation or generated force. If contraction of a muscle causes a joint to rotate, it will change the length of the muscle; if the muscle contraction does not result in rotation of a joint (e.g., due to the presence of external force) it may still change the muscle length. In either case, changes in muscle length could result in muscle afferent firing and such firing information could be fed back to excite motoneurons and produce EMG activity. We acknowledge these facts: that the joint motion we measured could be both a result and a cause of EMG activity and that a lesser or slower joint rotation does not immediately mean less Ia or II afferent firing or that these afferents do not contribute to EMG generation. Furthermore, not all afferent firing would result in motoneuron firing and muscle activation. For example, Prochazka & Gorassini (1998) showed that Ia afferents from the triceps surae fire most strongly during the swing phase of walking when the triceps are not active, indicating the separation of muscle activity and firing of its homonymous afferents in that specific phase of locomotion.

In examining neural control of walking in humans non‐invasively, we often need to use indirect measures and experimental manipulations. The data from invasive animal research studies are essential in interpreting such approximation or surrogate measures adequately, and the present study heavily relied on the previous findings from such animal studies (Appenteng & Prochazka, 1984; Donelan & Pearson, 2004a; Donelan et al., 2009; Hiebert et al., 1996; Houk & Henneman, 1967; Prochazka & Gorassini, 1998; Prochazka et al., 1989; Whelan et al., 1995).

### 4.9 Conclusion

In this study, we investigated how human adults manage additional task demands of going faster and carrying additional weight during walking. In walking faster, we found that there was a universal increase in burst EMG amplitude across flexors and extensors of upper and lower leg muscles while maintaining their burst patterns of activity, most of which are relatable to increased descending drive to the CPG (or CPG‐like spinal network for locomotion). At the same time, we observed a large increase in the mid–late stance phase plantarflexor activity with large increases in GRF measures, suggesting the contribution of force sensitive afferents to increasing propulsive force generating plantarflexor activity. When carrying additional weight, we found consistent increases in the extensor but not flexor activity, and the quadriceps activity in early stance was most notably increased with weight. While descending input to CPG likely remains important across different walking conditions, these results emphasize the afferent origin of such EMG activity modulation when carrying additional weight. When walking faster *and* carrying additional weight, changes in locomotor EMG and joint motion concerning the knee and hip joints displayed added‐up features of the two task demands, whereas plantarflexor activity and ankle joint motion did not appear to be a simple sum of the two, suggesting potential non‐linear interaction of different mechanisms across different walking speeds.

## AUTHOR CONTRIBUTIONS

Bridgette A. P. Damewood and Aiko K. Thompson contributed to conception and design of the study. Bridgette A. P. Damewood and Aiko K. Thompson contributed to the acquisition and analysis of the data. All authors contributed to the interpretation of the data. Bridgette A. P. Damewood and Aiko K. Thompson drafted the manuscript. All authors contributed to manuscript revision. All authors have read and approved the final version of this manuscript and agree to be accountable for all aspects of the work in ensuring that questions related to the accuracy or integrity of any part of the work are appropriately investigated and resolved. All persons designated as authors qualify for authorship, and all those who qualify for authorship are listed.

## CONFLICT OF INTEREST

None declared.

## Data Availability

The data supporting the conclusions of the current study will be made available by the corresponding author on reasonable request.
